# Prophylactic Potential of *Heyndrickxia coagulans* Strain LMG S-24828 in an In Vitro Model of ESBL–*Escherichia coli* Urothelial Infection

**DOI:** 10.3390/microorganisms14030606

**Published:** 2026-03-09

**Authors:** Luca Spaggiari, Natalia Pedretti, Muhammad Behzad, Ramona Iseppi, Carla Sabia, Maria Teresa Franzè, Carolina Cason, Rosario Russo, Manola Comar, Francesco De Seta, Andrea Ardizzoni, Eva Pericolini

**Affiliations:** 1 Department of Surgical, Medical, Dental and Morphological Sciences with Interest in Transplant, Oncological and Regenerative Medicine, University of Modena and Reggio Emilia, 41124 Modena, Italy; luca.spaggiari@unimore.it (L.S.); teresa.franze@unimore.it (M.T.F.); andrea.ardizzoni@unimore.it (A.A.); 2 Hip-Tech PhD Program, University of Modena and Reggio Emilia, 41125 Modena, Italy; natalia.pedretti@unimore.it (N.P.); 360343@studenti.unimore.it (M.B.); 3 Department of Life Sciences, University of Modena and Reggio Emilia, 41125 Modena, Italy; ramona.iseppi@unimore.it (R.I.); carla.sabia@unimore.it (C.S.); 4 Institute for Maternal and Child Health, IRCCS Burlo Garofolo, 34137 Trieste, Italy; carolina.cason@burlo.trieste.it (C.C.); manola.comar@burlo.trieste.it (M.C.); 5 Giellepi S.p.A., Via G. Verdi, 41/Q, 20831 Seregno, Italy; rosario.russo@giellepi.it; 6 Department of Clinical, Medical, Surgical and Health Sciences, University of Trieste, 34100 Trieste, Italy; 7 Department of Obstetrics and Gynecology, IRCCS San Raffaele Scientific Institute, University Vita and Salute, 20132 Milan, Italy; fradeseta@gmail.com

**Keywords:** *Heyndrickxia coagulans*, probiotics, *Escherichia coli*, T24 urothelial cells

## Abstract

Urinary tract infections (UTIs) are among the most common bacterial infections and represent a significant health concern worldwide. The most common cause of these infections is the Gram-negative bacterium *Escherichia coli* (*E. coli*), an opportunistic commensal of the human gut that can shift to pathogenicity, leading to a wide variety of diseases. The increasing ability of *E. coli* to develop resistance to various classes of antibiotics underscores the urgent need for alternative approaches to clear up infections caused by this species. In this study, we analyzed the possible beneficial role of *Heyndrickxia coagulans* (*H. coagulans*) strain LMG S-24828 in an in vitro model of T24 urothelial cells infection by ESBL-producing *E. coli*. Our results showed that *H. coagulans* LMG S-24828 was able to: (i) reduce *E. coli* growth; (ii) impair *E. coli* adhesion to T24 urothelial cells; and (iii) modulate cytokine production by T24 urothelial cells per se and after *E. coli* infection. Collectively, these findings indicate a beneficial effect of *H. coagulans* strain LMG S-24828 against an ESBL–*E. coli* isolate in an in vitro model of T24 urothelial cell infection.

## 1. Introduction

Bacterial urinary tract infections (UTIs) are a very common condition, since they affect more than 50% of women during their lifetime. Furthermore, up to 25% of such women may experience more than one episode per year, and the frequency may increase to two or more episodes within a 6-month period [1,2]. This condition, recurrent UTI (rUTI), poses a public health concern that involves economic, social, and personal aspects [3]. Uropathogenic strains (UPEC) of the species *Escherichia coli* (*E. coli*) are the main ones responsible for UTI and in particular the recurrent forms [2]. *E. coli* is a Gram-negative rod-shaped bacterium belonging to the *Enterobacteriaceae* family. It is an extremely versatile species, and it can survive and proliferate in a variety of natural and abiotic environments. It is abundant not only in the gut microbiota of humans (that colonizes within a few hours from birth), but also in the gut of other warm-blooded animals and reptiles, and it becomes one of the predominant species that dwell in the intestinal tract throughout the host’s life [4]. Under physiological conditions, the commensal relationship between *E. coli* and humans warrants the tolerance of the bacterium within the intestine. However, some strains of *E. coli* are responsible for human diseases. According to the clinical classification, these bacteria can be categorized into intestinal diarrhoeagenic strains (DEC) [5] and extra-intestinal pathogenic strains (ExPEC). At gut level, severe diarrheal or enteritis syndromes can be caused by *E. coli* DEC strains that acquire virulent traits and shift from commensalism to pathogenicity, and this phenomenon is due to genetic changes favored by the great plasticity of the *E. coli* genome [5]. Indeed, the versatile nature of *E. coli* and its capacity to adapt have allowed the spread of a very large number of strains with extremely varied genetic, physiological, and virulence characteristics. Furthermore, *E. coli* is able to translocate from the intestine to other body sites, causing a wide range of extraintestinal diseases such as UTIs, intra-abdominal, pulmonary, and skin infections, as well as neonatal meningitis and bacteremia and sepsis [6]. In addition, *E. coli* can colonize the genital tract and may contribute to vaginal dysbiosis and mucosal inflammation [7]. Beyond aerobic vaginitis, this opportunistic microorganism has been implicated in other genital tract infections, such as vulvovaginitis and endometritis, particularly in the presence of impaired local defense mechanisms [8]. These conditions are typically characterized by epithelial disruption, inflammatory response, and alterations of the normal vaginal microbiota. The ExPEC strains are endowed with specific surface molecules that allow the colonization of areas where they are not present under physiological conditions. Such molecules include type 1 fimbriae, P fimbriae, and numerous other adhesins that are expressed on the surface of the bacterial cells. The most common ExPEC strains are the uropathogenic strains (UPEC). Upon *E. coli* infection, the epithelial cells get damaged via the toxic activity of exotoxins and endotoxins. In fact, during evolution, the intestinal epithelium has undergone an adaptation that prevents the surface antigens of *E. coli* (such as lipopolysaccharide, LPS) from inducing a massive activation of the immune system. However, in body areas not physiologically colonized by this species, such adaptation has not occurred. Therefore, when the pathogen recognition receptors (PRRs) of the epithelial cells (including urothelial cells) perceive *E. coli*, they trigger the release of inflammatory signals in response to contact with the bacteria. It is important to point out that many strains of *E. coli* (including several UPEC strains) are able to shut down the pro-inflammatory response set off by epithelial cells or to mask their pathogen-associated molecular patterns (PAMPs) to evade the immune response [9]. *E. coli* infections are treated with antimicrobials. The latter contribute to the emergence of antimicrobial resistance, which is a major and increasing global healthcare problem [10]. In this context, the issue of drug-resistant *E. coli*, such as ESBL-producing *E. coli* (ESBL–*E. coli*), is causing significant alarm in human and veterinary medicine on a global scale. The World Health Organization (WHO) classifies *E. coli* as a critical priority pathogen due to its increasing antibiotic resistance [11] that poses a significant threat to public health and highlights the urgency of novel antimicrobial therapies. There is growing interest in the possibility of using probiotics in the treatment of urinary tract infections caused by *E. coli*. Most probiotics currently available on the market are typically Gram-positive bacteria belonging to the *Lactobacillus* and *Bifidobacterium* genera. However, in recent years, more evidence has emerged demonstrating the beneficial effects of other species. Among the latter, several spore-producing bacterial species stand out, in particular *H. coagulans* (formerly known as *Bacillus coagulans*). The ability to form spores provides a great advantage in the context of probiotics. The high resistance of spores, even to harsh conditions, allows the bacteria to survive the extreme acidity of the gastric environment and to reach the intestine alive. For the same reason, sporogenic bacteria can be freeze-dried, allowing probiotic formulations to be stored better, more easily, and for a longer time, and also facilitating their transport [12]. Among the various strains, the species *H. coagulans* is receiving increasing attention from researchers due its beneficial properties, especially on the intestinal tract, which have been described in numerous scientific studies. Specifically, *H. coagulans* has been shown to increase nutrient absorption by the intestinal mucosa, facilitating digestion and promoting peristalsis [13], to exert antimicrobial activity against various bacterial and fungal pathogens [14] and to maintain immune homeostasis through its immunomodulatory activity. In this work, we analyze the possible beneficial effect of *H. coagulans* strain LMG S-24828 in an in vitro model of human T24 urothelial cells infected by an ESBL–*E. coli* strain.

## 2. Materials and Methods

### 2.1. Microbial Strains and Growth Conditions

The *H. coagulans* strain LMG S-24828 (Weizy^®^) (Hc) was supplied by Giellepi S.p.A (Seregno, Italy) in the form of freeze-dried spores. Upon receipt, the spores were stored at 4 °C and germinated by incubation in tryptic soy broth (TSB) (Condalab, Madrid, Spain) for 24 h at 37 °C under agitation. In all the experiments, Hc cells in the exponential growth phase obtained from spore germination in TSB were used. Hc’s safety profile, including genomic analyses related to the absence of virulence and antimicrobial resistance determinants, has been recently described [15].

The *E. coli* strain used in the present study was isolated and identified by means of a Vitek^®^ 2 XL MS system (bioMérieux, Florence, Italy) from a fecal sample of heathy subject (therefore not associated with a urinary tract infection at the time of isolation) at the Institute for Maternal and Child Health IRCSS Burlo-Garofolo, Trieste, Italy. The study was conducted in accordance with the Declaration of Helsinki and approved by the Institutional Review Board of the Institute for Maternal and Child Health IRCSS Burlo-Garofolo, Trieste, Italy (protocol code 37902 dd. 10 December 2021). In selected experiments (cell-associated *E. coli* assay), *E. coli* ATCC strain 13762 was used. The strains were maintained by seeding on MacConkey agar (Sigma, Burlington, MA, USA), incubating at 37 °C for 24 h, and then storing at +4 °C for no more than two weeks. The *E. coli* strains were also stocked at −80 °C in cryovials containing plastic beads (Pro-Lab Diagnostics, Bromborough, UK). The *E. coli* broth culture used in the experiments was obtained by picking a colony, inoculating it in 5 mL of TSB, and incubating the broth at 37 °C under agitation for 24 h. To determine the bacterial concentration in the broth cultures, samples were centrifuged at 3500 rpm for 5 min. After removal of the supernatant, the bacterial pellet was resuspended in 1 mL of TSB. Then, 100 µL of bacterial suspension and 100 µL of sterile TSB (used as blank) were seeded in two wells of a 96-well plate (Sarstedt, Nümbrecht, Germany), and the optical density (OD) was measured at a wavelength of 595 nm using a spectrophotometer (Sunrise, Tecan, Männedorf, Switzerland). The OD of the blank was subtracted from the OD of the bacterial suspension, and the corresponding value of CFU/mL was calculated by interpolating the OD value to a previously established calibration curve.

### 2.2. E. coli Clinical Isolate Antimicrobial Profile

The *E. coli* clinical isolate antimicrobial susceptibility testing was conducted by means of the Vitek^®^ 2 XL MS system (bioMérieux, Florence, Italy). According to the EUCAST 2025 [16] antimicrobial clinical breakpoints, *E. coli* is classified as resistant (R), susceptible (S) or susceptible with increased exposure (IE) to the different antimicrobials tested. According to the combination of test results, including the synergy test, this strain was classified as ESBL-producing *E. coli*. ESBL production was tested by the double-disk synergy test (DDST), as recommended by the Clinical and Laboratory Standards Institute (CLSI) [17]. DDST was performed by placing discs of cefotaxime (CTX, 30 mg; Beckton, Dickinson and Company, Breda, Netherlands), cefotaxime with clavulanic acid (CTX-CLA, 30/10 mg), ceftazidime (CAZ, 30 mg), and ceftazidime with clavulanic acid (CAZ-CLA, 30/10 mg) on plates of Mueller–Hinton agar (MH, bioMérieux, Florence, Italy). ESBL production was considered positive when, following incubation at 37 °C for 24 h, the growth inhibitory zone, both around the CTX-CLA and the CAZ-CLA disks, was increased by 5 mm or more compared with the diameter around the disk containing CTX or CAZ alone. In addition, we performed an evaluation of *E. coli* clinical isolate susceptibility to CAZ by broth microdilution both with and without the addition of boronic acid using a MICRONAUT-S MDR MRGN screening panel (bioMérieux, Florence, Italy).

### 2.3. Effect of Hc on E. coli Clinical Isolate Growth

The impact of Hc on the growth of *E. coli* was assessed through co-incubation experiments at different combination ratios between the two bacterial species (multiplicity of infection, MOI). Specifically, MOIs (*E. coli*–Hc) of 1:100, 1:500, 1:1000, 1:5000, and 1:10,000 were tested. Five hundred microliters of TSB containing *E. coli* (1 × 10^8^ CFU/mL) was dispensed into the wells of a 24-well plate (Sarstedt, Nümbrecht, Germany), followed by the addition of 500 µL of TSB containing Hc at the appropriate concentrations. As a control sample, 500 µL of sterile TSB were added to a well containing *E. coli*. The plate was then incubated at 37 °C with 5% CO_2_ for 24 h. After incubation, the supernatant from each sample was collected, and 500 µL of soybean–casein–digest–lecithin–polysorbate 80 (SCDLP80, Biotec, Dueville, Italy) broth was added to each well. After pipetting vigorously to detach the bacterial cells adhering to the plastic surface, the contents of each well were removed and combined with the corresponding previously collected supernatant. The samples were serially diluted and plated on CHROMagar^TM^ *E. coli* (Biolife, Milan, Italy) to exclude the growth of Hc colonies. The plates were incubated at 37 °C for 24 h, and the colony forming units (CFUs) were determined by counting the blue colonies.

### 2.4. Human T24 Urothelial Cells

The T24 cell line (Cell Line Service, 300352), derived from urothelial carcinoma, was used in this study. Cells were cultured in DMEM–Ham’s F12 medium (Cell Line Service, Braunschweig, Germany) supplemented with penicillin (100 U/mL) (Euroclone S.p.a., Pero, Italy), streptomycin (100 µL/mL) (Euroclone S.p.a., Pero, Italy), ciprofloxacin (20 mg/mL) (Euroclone S.p.a., Pero, Italy), and FBS (fetal bovine serum, 10% or 5%, Sigma-Aldrich, St. Louis, MO, USA). The cell line was kept viable by subculturing twice a week and incubating at 37 °C with 5% CO_2_.

### 2.5. Generation of a Confluent Monolayer of Human T24 Urothelial Cells

For all the experiments involving T24 urothelial cells, a confluent monolayer was prepared. After removing the culture medium, the cells were washed with pre-warmed PBS (phosphate-buffered saline, Sial, Roma, Italy) and detached by incubation in 0.25% trypsin–EDTA solution for 90 s. Once detached, trypsin activity was neutralized by adding complete culture medium, and the cell suspension was collected and centrifuged at 500 rpm for 5 min. Once the supernatant had been discarded, the cell pellet was resuspended in medium supplemented with 10% FBS. The cell concentration was determined by a Bürker chamber under an optical microscope and excluding dead cells by staining with trypan blue (Corning Inc., Corning, NY, USA). Subsequently, 1 mL of cell suspension containing 5 × 10^5^ cells was seeded into the wells of a 24-well plate (Greiner Bio-One, Kremsmünster, Austria) and incubated at 37 °C with 5% CO_2_ for 48 h to allow the formation of a confluent monolayer. Prior to being used for the experiments, the cell monolayer was washed very gently with PBS, and the culture medium was replaced with 1 mL of fresh antibiotic-free medium supplemented with 5% FBS.

### 2.6. Establishment of the In Vitro Infection Model

To determine the highest concentration of Hc tolerated by a urothelial cell monolayer after 24 h of colonization (i.e., the maximum bacterial concentration capable of not causing damage to urothelial cells), different concentrations of Hc were added to a confluent monolayer of T24 urothelial cells. The plate was then incubated at 37 °C with 5% CO_2_ for 24 h. Specifically, the following MOIs (urothelial cells–Hc cells) were tested: 1:10, 1:50, 1:100, and 1:250. At the end of incubation, cell damage in each sample was quantified using a lactate dehydrogenase (LDH) assay employing a specific commercially available kit (Hoffmann-La Roche, Basel, Switzerland) and following the manufacturer’s instructions. Since—as previously described [14]—Hc produces organic acids that lower the pH of culture medium (at about pH 4.4) and can thus impair LDH enzyme activity, an alternative protocol was applied to directly quantify LDH levels in the culture medium [18]. Briefly, after removing the culture medium, the cells were washed very gently with 1 mL of pre-warmed Hank’s balanced salt solution (HBSS, Gibco, Grand Island, NY, USA). The cells were then lysed by adding 1 mL of 0.2% Triton X-100 (Sigma Aldrich, St. Louis, MO, USA) and incubating for 5 min before vigorous pipetting. Finally, 100 µL of each sample was analyzed using the LDH assay.

This assay is based on a quantitative colorimetric reaction, where the intensity of the color production is proportional to the LDH concentration in the sample. Color development was quantified by spectrophotometric measurement of the OD at a wavelength of 492 nm (with 620 nm as the reference wavelength). As a control sample, cells not colonized by Hc were analyzed, and 100% cell viability was assigned to the OD value obtained from this sample. Accordingly, the percentage of viable cells in each sample was calculated by proportion, while the percentage of cell death was determined as the complementary value.

Moreover, to establish the infection model, the lowest concentration of *E. coli* clinical isolate capable of causing extensive damage to the urothelial cell monolayer after 6 h of infection was determined. The T24 cell monolayer was infected with 1 mL of culture medium containing different concentrations (MOI 1:1, 1:10, 1:100, and 1:1000) of *E. coli* clinical isolate and then incubated at 37 °C with 5% CO_2_ for 6 h. At the end of the incubation, the cell damage was quantified using the LDH assay, as described above.

### 2.7. Effect of Hc on the Cell-Association Capacity of E. coli with a Monolayer of T24 Urothelial Cells

To evaluate the effect of Hc on the ability of *E. coli* (clinical isolate and ATCC strain) to associate with T24 urothelial cells, 500 µL of culture medium containing Hc (MOI 1:100, 5 × 10^7^ Hc cells) was added to a confluent monolayer of T24 urothelial cells. The plate was then incubated at 37 °C with 5% CO_2_ for 3 h. Then, 500 µL of *E. coli* suspension (MOI 1:100, 5 × 10^7^ bacterial cells) was added to each well, and the samples were incubated at 37 °C with 5% CO_2_ for a further 3 h. After incubation, the culture medium was removed, and the wells were gently washed with 1 mL of warm PBS to remove non-adherent bacteria. The cell monolayers were then lysed by adding 1 mL of 0.2% Triton X-100 followed by vigorous pipetting. The resulting suspensions were serially diluted and plated on CHROMagar^TM^ *E. coli*. The plates were incubated at 37 °C for 24 h, and blue colonies were counted to determine the concentration of *E. coli* associated with the urothelial cells. A monolayer of T24 cells infected with *E. coli* not pre-colonized with Hc was used as a control sample.

### 2.8. Cytokine Production After E. coli Clinical Isolate Infection in the Presence of Hc

The release of interleukin (IL)-1α, IL-1β, IL-6, and IL-8 by T24 urothelial cells pre-colonized with Hc for 18 h (urothelial cells–Hc, MOI 1:100) and subsequently infected or not with *E. coli* clinical isolate for an additional 6 h (urothelial cells–Hc–*E. coli*, MOI 1:100:100) was quantified using commercial ELISA kits (IL-1 α , IL-6: PeproTech, Thermo Fisher Scientific, Waltham, MA, USA; IL-1 β , IL-8: Invitrogen, St. Louis, MO, USA) according to the manufacturers’ instructions.

### 2.9. Statistical Analysis

Statistical analyses were performed using GraphPad Prism 10 version 10.4.0 (San Diego, CA, USA). The normality of data distribution was assessed using the Shapiro–Wilk test. Data with a normal (Gaussian) distribution were analyzed using either an unpaired two-tailed Student’s *t*-test or ordinary one-way ANOVA followed by an uncorrected Fisher’s LSD multiple comparison test. Data that did not follow a normal distribution were analyzed using the Kruskal–Wallis test followed by uncorrected Dunn’s multiple-comparison test or the Mann–Whitney U test when comparing two groups. The specific statistical test applied to each analysis is indicated in the corresponding figure caption. Data shown in the graphs are from at least three independent experiments. Values of *p* < 0.05 were considered statistically significant. Reductions are indicated by a hash symbol (#), and increases are indicated by an asterisk (*).

## 3. Results

### 3.1. E. coli Clinical Isolate Antibacterial Susceptibility Test

The antibacterial susceptibility profile of the *E. coli* clinical isolate was tested. The data reported in Table 1 show that *E. coli* is resistant to amoxicillin–clavulanic acid (CLA), cefotaxime (CTX), and cefepime and has intermediate resistance to cefoxitin and ceftazidime (CAZ). The synergy test revealed an increase in halo diameter of >5 mm for both CTX–CLA and CAZ–CLA compared to CTX and CAZ alone. Therefore, these results confirmed that this strain is ESBL-producing *E. coli* (ESBL–*E. coli*). Moreover, we performed MICRONAUT-S MDR MRGN screening to exclude the possibility that this *E. coli* strain was an AmpC producer. Our results showed that the MIC in the presence of boronic acid was identical to that of CAZ alone, indicating the absence of AmpC activity.

### 3.2. Hc Impairs E. coli Clinical Isolate Growth

**Figure 1:**
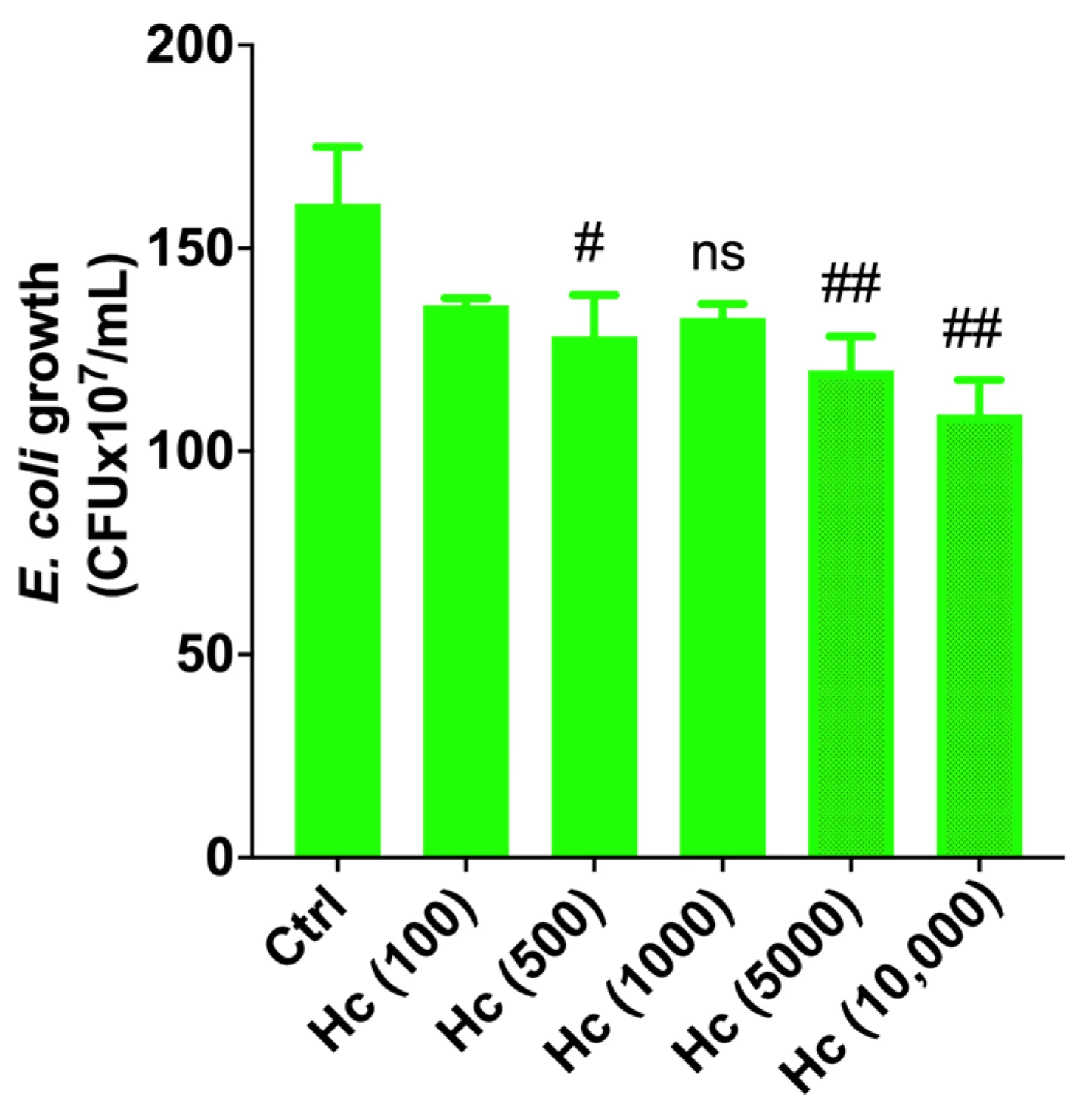
Effect of Hc on *E. coli* growth. *E. coli* growth was quantified after 24 h of co-culture at 37 °C with Hc at different MOI (*E. coli*-Hc, MOI 1:100, 1:500, 1:1000, 1:5000, 1:10,000). The graphs show the means ± SEM of CFU × 10^7^/mL from 6 independent experiments (ctrl and Hc (5000) was tested in 6 experiments; Hc (500) was tested in 5 experiments; Hc (1000) and Hc (100) was tested in 4 experiments; Hc (10,000) was tested in 3 experiments). Statistical analysis was performed using ordinary one-way ANOVA followed by uncorrected Fisher’s LSD multiple comparison tests. ns = not significant; ^#^ *p* < 0.05; ^##^ *p* < 0.01.

*E. coli* is the main causative agent of urinary tract infections; therefore, the ability of Hc to affect *E. coli* clinical isolate growth is considered the first parameter to rate Hc effectiveness. Indeed, the antimicrobial activity against pathogens is a distinctive feature of probiotic microorganisms [19]. To this end, *E. coli* was incubated with increasing concentrations of Hc (*E. coli*–Hc, MOI 1:100, 1:500, 1:1000, 1:5000, 1:10,000) at 37 °C for 24 h. Then, *E. coli* growth was assessed by CFU counting on CHROMagar^TM^ *E. coli*. The results showed that Hc significantly inhibited *E. coli* growth starting from MOI 1:500 with a higher reduction from MOI 1:5000 (Figure 1).

### 3.3. Establishment of the In Vitro Urothelial Cell Infection Model

**Figure 2:**
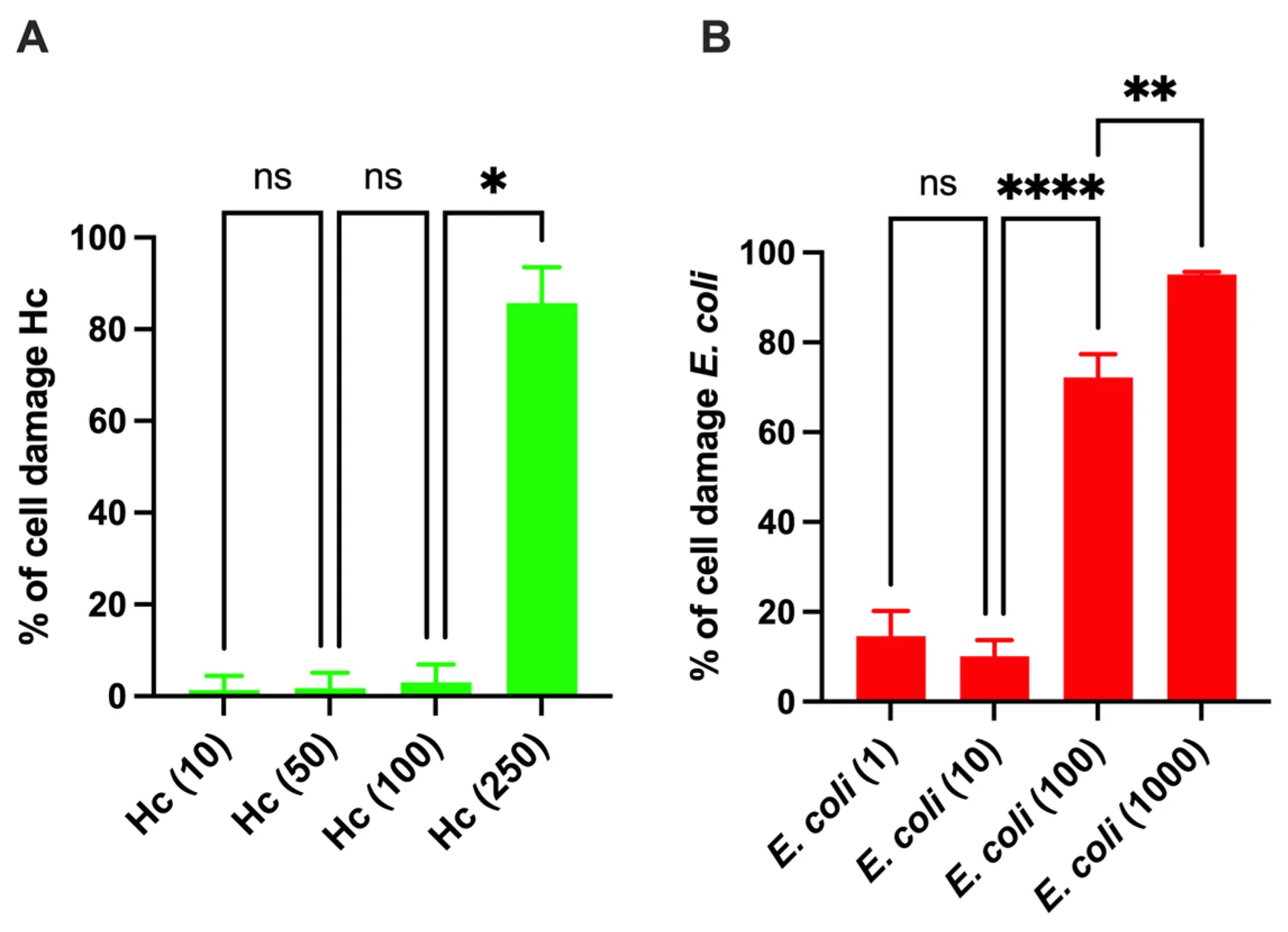
(**A**) Urothelial cell damage after cell monolayer colonization with different concentrations of Hc (urothelial cells–Hc 1:10, 50, 100, and 250) for 24 h. The graph shows the means ± SEM of the percentage of cell damage from 5 independent experiments. Statistical analysis was performed using Kruskal–Wallis tests followed by uncorrected Dunn’s multiple-comparison tests. (**B**) Urothelial cell damage induced by different concentrations of *E. coli* after 6 h of infection (urothelial cells–*E. coli* 1:1, 10, 100, and 1000). The graph shows the mean % ± SEM of cell damage from 3 independent experiments. Statistical analysis was performed using ordinary one-way ANOVA followed by uncorrected Fisher’s LSD multiple-comparison tests. * *p* < 0.05; ** *p* < 0.01; **** *p* < 0.0001; ns = not significant.

Given that Hc exerts an anti-*E. coli* effect, to contextualize the experiments within the field of urinary tract infections, an in vitro infection model consisting of a confluent monolayer of human urothelial cells was employed. We first determined the optimal bacterial concentrations to be used in such infection model. Concerning Hc, the highest bacterial concentration tolerated by urothelial cells, unable to induce cell damage after 24 h of colonization, was determined. The results showed that up to a concentration of MOI 1:100 (urothelial cells–Hc), Hc was well tolerated by the urothelial cells (Figure 2A). At MOIs higher than 1:100 (specifically, from MOI 1:250), extensive cell damage occurred. Indeed, microscopic observation revealed marked alterations in cell morphology, accompanied by loss of cell adhesion and subsequent detachment. The observed phenotypes are consistent with severe structural alterations of the cell membrane. Therefore, the MOI 1:100 was chosen for the subsequent experiments.

As for the *E. coli* clinical isolate, the lowest bacterial concentration capable of inducing significant damage to the cell monolayer after 6 h of infection was investigated. As shown in Figure 2B, at MOIs equal to or greater than 1:100 (urothelial cells–*E. coli*), severe cytotoxicity was observed. Specifically, at MOI 1:100, *E. coli* caused an average cell death of 72.2%, which led us to select this concentration for subsequent experiments. Consequently, in the urothelial cell infection model, a urothelial cell–Hc–*E. coli* = 1:100:100 ratio was chosen.

**Figure 3:**
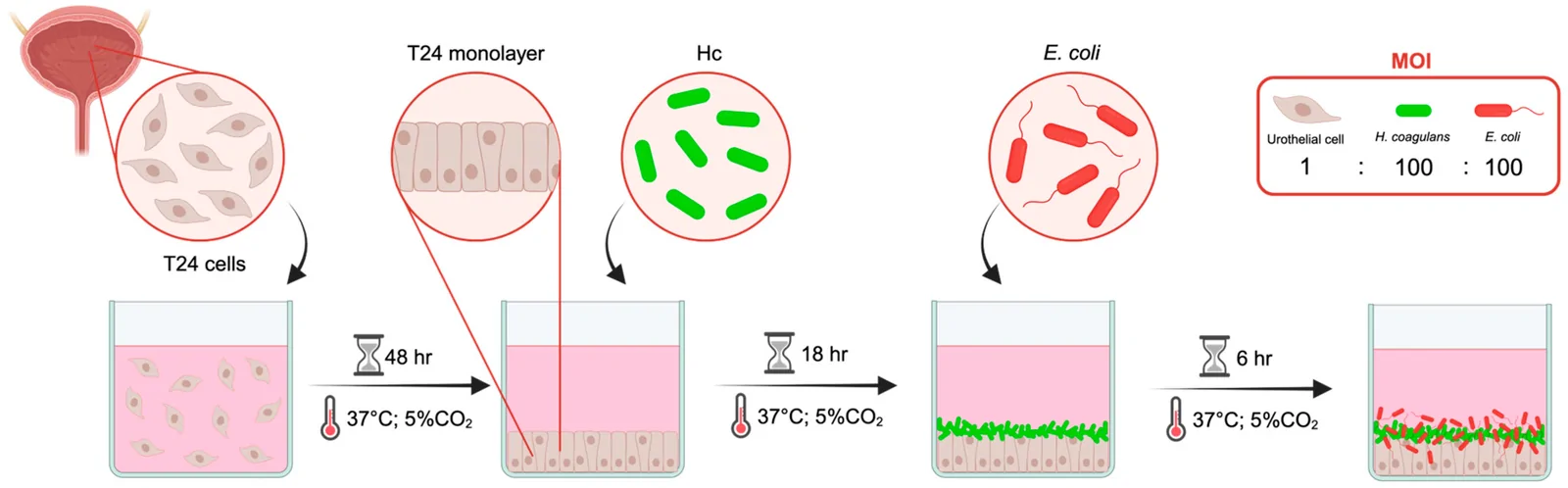
Schematic representation of the in vitro protocol employed for the infection of T24 urothelial cells. Created in BioRender. Ardizzoni, A. (2026) https://BioRender.com/9woovw0 (accessed on 4 March 2026).

A schematic representation of the in vitro urothelial cell infection protocol is depicted in Figure 3. In this in vitro infection model, the *E. coli*–Hc ratio (100:100) differed substantially from the ratio employed to analyze the direct antibacterial effect of Hc on *E. coli* growth (range of *E. coli*–Hc ratio: 1:100 to 1:10,000, Figure 1). This adjustment was necessary to preserve urothelial integrity following colonization with Hc for up to 24 h.

For these reasons, the in vitro urothelial infection model was used to investigate additional effects exerted by Hc after colonization of urothelial cells unrelated to its direct inhibition of *E. coli* growth (that was shown to start from an *E. coli*–Hc ratio 1:500, as shown in Figure 1), such as the immunomodulatory effect per se and during *E. coli* infection.

### 3.4. Effect of Hc on Cell-Associated E. coli to Urothelial Cells

**Figure 4:**
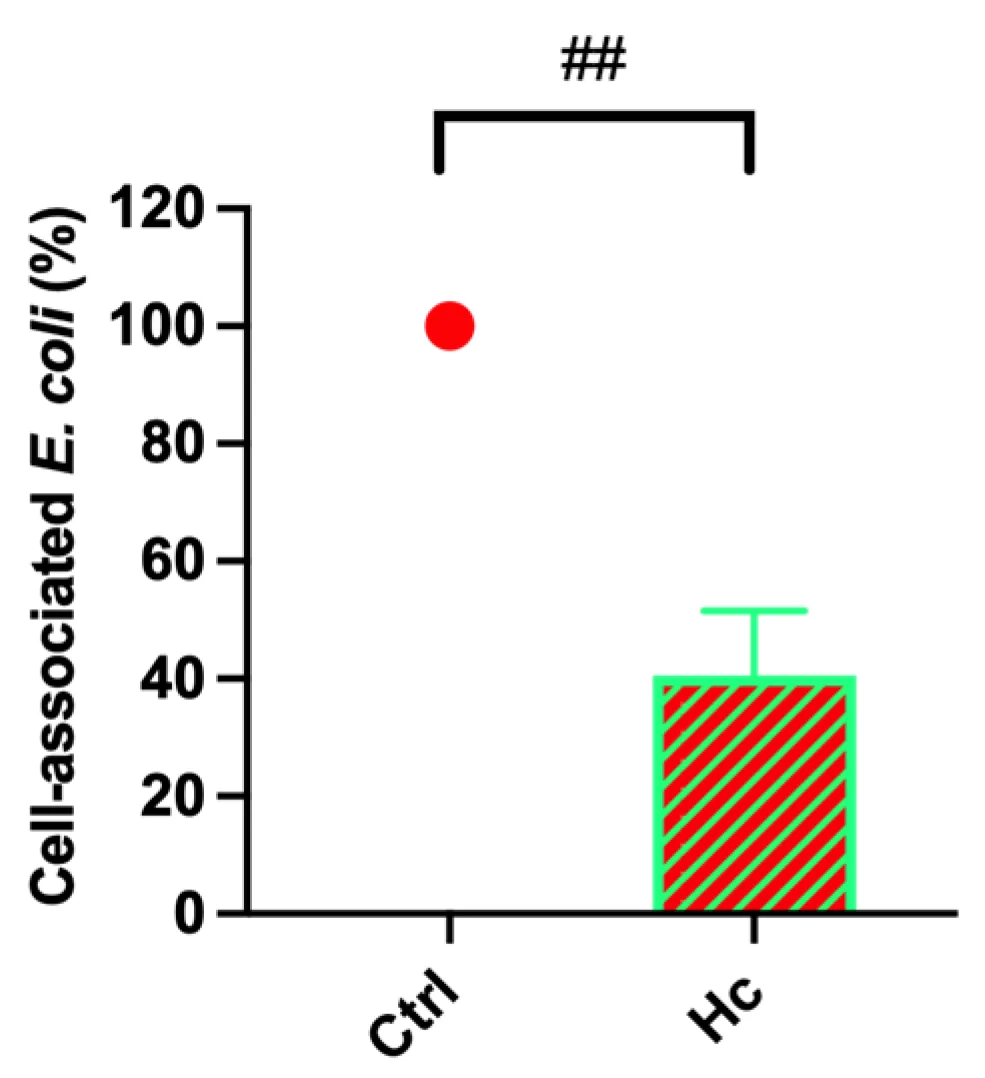
Effect of pre-colonization with Hc on *E. coli* associated with T24 urothelial cell monolayer. The graph shows the mean percentage ± SEM of *E. coli* associated with urothelial cells, calculated by establishing the number of adhered *E. coli* in the control samples (Ctrl) as 100%. The data reported are from 3 independent experiments. Statistical analysis was performed using unpaired Student’s *t*-test. ^##^ *p* < 0.01.

We next evaluated whether pre-colonization of urothelial cells with Hc could affect the ability of *E. coli* to associate with them. The capacity of *E. coli* to adhere is a critical virulence factor of this species, and it plays a crucial role in the early stages of the infectious process. Indeed, *E. coli* must firmly adhere to the epithelium of the urinary tract to avoid being flushed out by the normal flow of urine [20]. To this end, a confluent monolayer of urothelial cells was pre-colonized with Hc for 3 h. Subsequently, *E. coli* was added and samples were further incubated for 3 h. Next, the cell monolayers were gently washed to remove non-adherent bacteria, and the fraction of viable cell-associated *E. coli* was quantified as detailed in the Section 2. The results showed that pre-colonization of urothelial cells with Hc led to a significant reduction in the amount of *E. coli* clinical isolate associated with the cell monolayer (Figure 4 and Supplementary Figure S1). We also compared the cell-association capacity of *E. coli* clinical isolate with the *E. coli* ATCC strain 13762 with the same experimental settings. No significant differences in cell-association capacity were observed between the two strains (Supplementary Figure S2). Notably, for the ATCC strain also, Hc pre-colonization led to a significant reduction in the percentage of *E. coli* associated with the T24 cell monolayer (Supplementary Figure S3).

### 3.5. Immunomodulatory Properties of Hc

**Figure 5:**
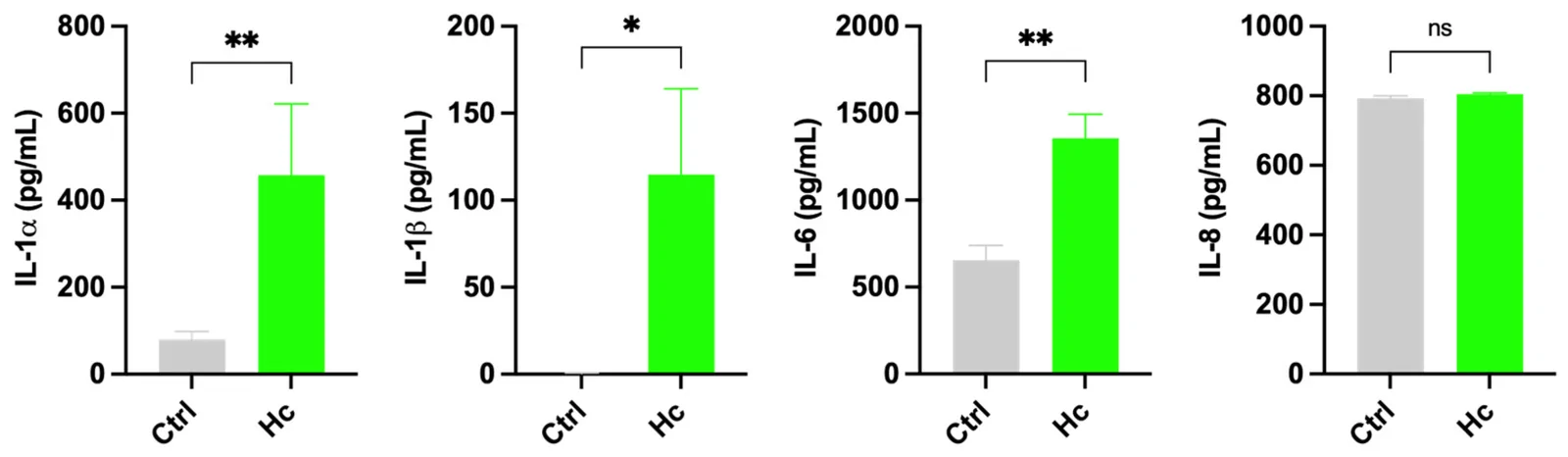
Immunomodulatory effect of Hc. Cytokine production from urothelial cells colonized for 24 h with Hc (urothelial cells–Hc 1:100). The levels of IL-1α, IL-1β, IL-6 and IL-8 were measured in culture supernatants. Graphs show mean pg/mL ± SEM from 4 independent experiments (IL-1β and IL-8) and from 5 independent experiments (IL-1α and IL-6). Statistical analysis was performed using unpaired Student’s *t*-test (IL-6 and IL-8) or Mann–Whitney U test (IL-1α and IL-1β). * *p* < 0.05; ** *p* < 0.01; ns = not significant.

To evaluate the intrinsic immunomodulatory properties of Hc per se, cytokine release by urothelial cells monolayer was evaluated after 24 h of Hc colonization. Our results showed that Hc significantly increased the basal-level production of IL-1α, IL-1β, and IL-6 by T24 urothelial cells, whereas it did not alter IL-8 secretion, whose basal level was already high (Figure 5).

**Figure 6:**
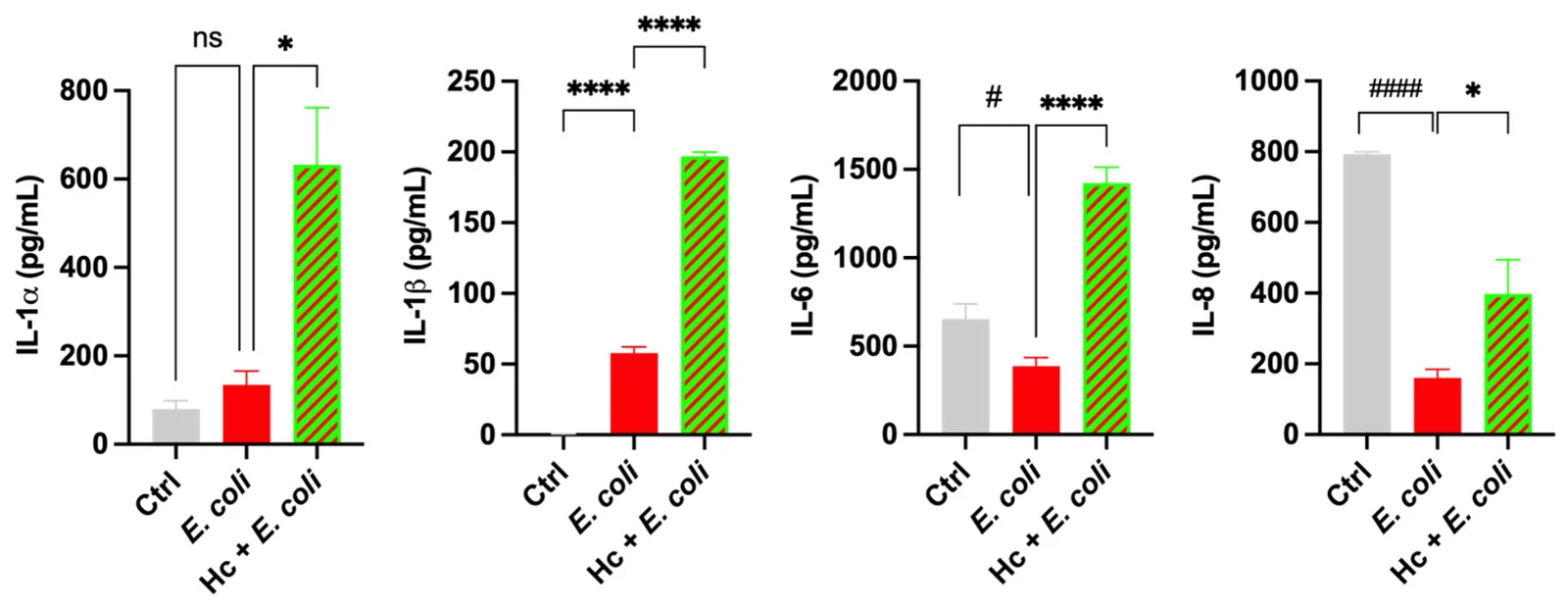
Immunomodulatory effect of Hc pre-colonization after infection with *E. coli*. Cytokine production from urothelial cells pre-colonized for 18 h with Hc and then infected for 6 h with *E. coli*. After 24 h of co-culture (urothelial cells–Hc–*E. coli* 1:100:100), IL-1α, IL-1β, IL-6, and IL-8 were quantified from culture supernatants. Graphs show the mean pg/mL ± SEM from 4 independent experiments (IL-1β and IL-8) and from 5 independent experiments (IL-1α and IL-6). Statistical analysis was performed using ordinary one-way ANOVA followed by uncorrected Fisher’s LSD (IL-1β, IL-6 and IL-8) or Kruskal–Wallis tests followed by uncorrected Dunn’s multiple-comparison tests (IL-1α). * *p* < 0.05; **** *p* < 0.0001; ^#^ *p* < 0.05; ^####^ *p* < 0.0001; ns = not significant.

Next, we analyzed cytokine release after infection with *E. coli* for 6 h by urothelial cells that had been pre-colonized by Hc. Our results show that *E. coli* infection significantly increased IL-1β production and decreased both IL-6 and IL-8 production compared to uninfected urothelial cells. In contrast, *E. coli* infection did not modify IL-1α secretion by urothelial cells from its basal level. The pre-colonization of urothelial cells with Hc significantly enhanced the production of all the tested cytokines in *E. coli*-infected urothelial cells compared to non-pre-colonized cells infected with *E. coli* alone. These findings highlight the remarkable immunomodulatory capacity of Hc on urothelial cells (Figure 6).

## 4. Discussion

Six to eight million cases of urinary tract infections (UTIs) are reported each year in the USA, which makes them among the most common bacterial infections. Because of their anatomical characteristics, women are especially susceptible to UTIs, since bacteria can easily reach the urethra and the bladder by ascending from the perineal region. Among all the bacteria potentially responsible for such infections, the species *E. coli*, in particular the uropathogenic strains (UPEC), account for up to 80% of all the UTIs. This is due to specialized bacterial structures, such as type 1 fimbriae and P fimbriae, that allow the bacteria to adhere, colonize, and persist on the epithelial cells of the urinary tract [21]. The ability of UPEC to adhere to the epithelial cells of the urinary tract is a crucial virulence trait that allows the infection to establish and progress [22,23,24,25]. UPEC type 1 fimbriae D-mannose-specific adhesin (FimH) binds to the uroplakin 1a (UP1a) receptor, which is expressed on the surface of urothelial cells. The binding occurs through the terminal epitopes of UP1a-conjugated highly mannosylated glycans [26,27,28,29,30,31]. It has been demonstrated that FimH-mediated bacterial adhesion can be inhibited by D-mannose and its analogues through a competitive mechanism. In accordance, the employment of D-mannose to treat UTIs has been exploited by food and pharmaceutical industries for medical purposes [32,33]. Recent evidence suggests that despite being extracellular pathogens, UPEC strains are able to enter the host cells, which grants them a further virulence trait that facilitates escape from host immune surveillance. The first-line therapeutic treatment of UTIs still relies on antibiotics, even though the ever-increasing number of resistance strains, including those that express extended-spectrum β-lactamases, is posing a severe problem in the treatment and management of UTIs and requires the establishment of alternative or complementary approaches [34,35].

Because of the increasing evidence on the potential of probiotics to inhibit the growth of pathogens and to modulate host immune responses, by means of an in vitro infection model, we showed that *H. coagulans* strain LMG S-24828 (Hc) has the capacity to reduce *E. coli* association with urothelial cells. In our model, we employed an ESBL-producing *E. coli* strain with a multidrug-resistant profile as a prototype of those *E. coli* strains responsible for difficult-to-treat UTIs and for which alternative therapeutic strategies are warranted. We showed that Hc is able to reduce *E. coli* growth in a dose-dependent manner, achieving a statistically significant growth reduction at an *E. coli*–Hc ratio of 1:500, with higher and significant reduction from 1:5000. In order to preserve the integrity of the urothelial monolayer upon Hc colonization, the model was designed to keep a urothelial cell–Hc ratio of 1:100. Notwithstanding that at such a ratio the Hc is well below the ratio where it exerts an antibacterial effect, in this context our goal was to observe only the cell-stimulation effect of Hc. Nevertheless, we may envisage that in the context of ongoing treatment, both effects could be valid due to increased Hc concentration in the area. Furthermore, in order to have at least 70% cell damage after 6 h of infection, a 1:100 urothelial cells–*E. coli* ratio was chosen.

With this protocol, we assessed the release of cytokines by urothelial cells, and we have shown that Hc is able to increase cytokine production per se and in response to *E. coli* infection. Indeed, the urothelial cell secretion of (and response to) cytokines and chemokines is part of the reaction to UTIs [36]. Specifically, by treating urothelial cells with Hc, significant increases in secretion of IL-1α, IL-1β, and IL-6 was detected, whereas IL-8 remained as high as at its basal level. In line with these results, we also showed that upon *E. coli* infection, only the production of IL-1β increases, which mirrors literature data that report an increase of IL-1β mRNA levels in T24 cells infected by uropathogenic *E. coli* strains [37]. Conversely, IL-1α levels remained similar to those of uninfected cells. Although Hc did not exhibit an anti-inflammatory profile under the experimental conditions employed, the upregulation of IL-1α, IL-1β, and IL-6 should not be interpreted as evidence of intrinsic pro-inflammatory activity, but rather as the capacity to activate an efficient urothelial response to *E. coli*. These findings suggest that Hc may subtly modulate the basal activation state of innate immune pathways. Indeed, cytokines belonging to the IL-1 family are key regulators of early host defense, and small variations in their basal expression levels are known to fine-tune immune responsiveness rather than initiating inflammation per se. In this context, the observed cytokine changes are consistent with a priming effect whereby innate immune cells are maintained in a prompted state without undergoing autonomous inflammatory activation. This interpretation is further supported by the response to *E. coli* challenge. The increased cytokine production observed in Hc-pretreated cells became apparent following bacterial exposure, a condition known to activate pattern recognition receptors (PRRs), such as TLR4 [38]. Concerning IL-1β, the stimulation of TLR4 by Hc initiated a two-step process for IL-1β secretion, including a priming effect (via NF-kB activation and expression of pro-IL-1β and NLRP3) and a second step via NLRP3 inflammasome assembly, activation of caspase 1, and cleavage of pro-IL-1β into its active, secreted form [39]. The interaction with PRRs can be mediated by Hc microbial structures and/or metabolic products, leading to urothelial cells priming, a process known as training immunity [40]. The mechanistic basis of urothelial cell priming by Hc will be investigated in future work. Accordingly, the amplified cytokine response elicited by *E. coli* in Hc-pretreated cells likely reflects an increased sensitivity or lowered activation threshold of innate immune response, rather than a shift toward a constitutively pro-inflammatory phenotype. This primed state may enable a more rapid and efficient response to bacterial challenge, thereby supporting host defense mechanisms. Taken together, these data suggest that Hc promotes a controlled pre-activation of innate immunity, enhancing cellular responsiveness to microbial stimuli. It should be noted that host response varies considerably depending on the *E. coli* strain, and significant differences in host-response mechanisms have been identified when host cells were stimulated by ESBL- or non-ESBL-producing *E. coli* strains in in vitro studies [41]. Demirel I. and coworkers showed that in both ESBL- or non-ESBL-producing *E. coli* groups, there are strains that fail to induce IL-6 and IL-8, but notably, among strains that were able to active cytokines, cytokine levels were always higher in cells infected by non-ESBL strains, suggesting that ESBL–*E. coli* strains are more prone to evading the immune response [41]. Albeit with the limitation of an in vitro study performed with a single ESBL–*E. coli* strain, we suggest that some strains of *E. coli* (particularly the ESBL producers) may use the increased ability to evade the immune response as a virulence mechanism. In this context, a probiotic with the potential to improve the cellular response might have a beneficial effect against specific strains of *E. coli*. It should be pointed out that the efficacy of Hc might vary depending on the *E. coli* strain. While further studies are required to elucidate the functional consequences of this primed immune profile, the present findings indicate that Hc modulates immune reactivity in a context-dependent manner, shaping the magnitude and kinetics of cytokine release during bacterial challenge rather than driving chronic inflammatory signaling. In addition, after exposure to *E. coli*, urothelial cells rapidly increase IL-6 secretion [42,43,44], which is elicited by bacterial components such as lipopolysaccharide (LPS) and P-fimbriae [45,46,47]. Unlike laboratory strains, UPEC strains have been shown to suppress IL-6 secretion by urothelial cells in vitro, and this mechanism has been suggested to be used by these strains to escape host immune response and thus to survive [48,49,50,51]. The correlation between IL-6 production and UTI severity has also been demonstrated in in vivo models [52,53]. Specifically, a model of complicated UTI associated with urethral obstruction due to transurethral UPEC inoculation showed that the mortality rate was higher in IL-6-knockout (KO) mice than controls. In addition, in the IL-6-KO survivors, higher renal UPEC burden and worse renal inflammation was observed [54]. IL-8 is a potent chemoattractant that plays a pivotal role in neutrophil recruitment, and it is also secreted by urothelial cells in vitro. In mice, knockout of the IL-8 receptor causes a dysfunctional response of neutrophils to *E. coli,* UTIs, and renal scarring. The levels of IL-8 secreted by urothelial cells are influenced by *E. coli* fimbriae [55,56,57]. Therefore, we can hypothesize that cytokine modulation by urothelial cells is closely linked to strain-specific virulence factors of *E. coli*. In line with these data, in our in vitro infection model, *E. coli* was able to significantly downregulate the basal-level secretion of both IL-6 and IL-8, hence impairing the response of urothelial cells. However, upon pretreatment of urothelial cells with Hc, the downregulation of IL-6 was neutralized and the downregulation of IL-8 reduced.

In conclusion, although UPEC-associated virulence factors were not assessed for the strain of *E. coli* used in the present work, our data demonstrate that Hc impairs *E. coli* growth and reduces the capacity of the bacterium to associate with urothelial cells. In addition, Hc acts also as a priming signal for urothelial cells, promoting a basal positive activation of urothelial cells that prepares them for subsequent *E. coli* infection and potentiates the effectiveness of their response in a way that resembles a training immunity effect. A limitation of the present study is that the in vitro T24 urothelial cell infection model, despite being widely used to analyze urothelial response to *E. coli* infection, cannot faithfully reproduce the in vivo urothelial environment and may present a different expression profile of PRRs compared to primary urothelial cells. Consequently, the effect of Hc in the context of UTI will be validated in vivo. This include pre-clinical and clinical studies that can determine the ability, following oral administration of Hc, to colonize the urothelial cells and exert a preventive effect against *E. coli.* Moreover, a broader analysis of our in vitro system with more *E. coli* clinical isolates should be carried out to strengthen our data. Since the onset of UTIs is often the consequence of UPEC capacity to escape local immune responses, the results described here suggest that the beneficial effects of *H. coagulans* LMG S-24828 against *E. coli* in our in vitro model of urothelial cell infection are mediated by the capacity of Hc to promote a basal positive activation of urothelial cells, potentiating the effectiveness of their response to *E. coli* infection. Here, we extended our previous work delineating the beneficial effect of Hc in the context of vaginal infection by *Candida* spp. [14] by describing new activity of Hc against *E. coli*. This is not only direct antimicrobial activity, but also the Hc’s capacity to prime urothelial cells to respond more actively to highly virulent strains of *E. coli*, which often exploit their ability to evade the immune response as a virulence mechanism. This new finding, once supported by clinical evidence, may open new prospects for the prevention and management of urogenital infection sustained by *E. coli*.

## Figures and Tables

**Table 1:** Antimicrobial profile of *E. coli* clinical isolate.

Antimicrobial	MIC (μg/mL)	Reading
Amoxicillin–clavulanic acid	32	R
Piperacillin–tazobactam	≤4	S
Cefoxitin	≤4	IE
Cefotaxime	≥64	R
Ceftazidime	2	IE
Cefepime	16	R
Ertapenem	≤0.12	S
Meropenem	≤0.25	S
Amikacin	2	S
Gentamicin	≤1	S
Ciprofloxacin	≤0.06	S
Levofloxacin	≤0.12	S
Fosfomycin	≤16	S
Colistin	0.5	S
Trimethoprim–sulfamethoxazole	≤20	S

Breakpoints EUCAST 2025; S = susceptible; R = resistant; IE = susceptible to increased exposure.

## Data Availability

The original contributions presented in this study are included in the article/Supplementary Material. Further inquiries can be directed to the corresponding author.

## Supplementary Materials

The following supporting information can be downloaded at: https://www.mdpi.com/article/10.3390/microorganisms14030606/s1, Supplementary Figure S1. Effect of pre-colonization with Hc on *E. coli* (clinical isolate) associated to T24 urothelial cells monolayer. The graph shows the mean CFUx10^5^/mL ± SEM of *E. coli* associated to urothelial cells. The data reported are from n = 3 independent experiments. Statistical analysis was performed using unpaired Student’s *t*-test. ^#^ *p* < 0.05. Supplementary Figure S2. *E. coli* association capacity to T24 urothelial cells monolayer. The graph shows the mean CFUx10^5^/mL ± SEM of *E. coli* (ATCC strain and clinical isolate) associated to urothelial cells. The data reported are from n = 4 independent experiments (*E. coli* ATCC) and n = 3 independent experiments (*E. coli* clinical isolate). Statistical analysis was performed using unpaired Student’s *t*-test. Supplementary Figure S3. Effect of pre-colonization with Hc on *E. coli* ATCC strain associated to T24 urothelial cells monolayer. The graph shows the mean % of *E. coli* associated to urothelial cells. The data reported are from n = 4 independent experiments. Statistical analysis was performed using unpaired Student’s *t*-test. ^###^ *p* < 0.001.
