# Supplementary material for: Prophylactic Potential of Heyndrickxia coagulans Strain LMG S-24828 in an In Vitro Model of ESBL–Escherichia coli Urothelial Infection
Source: Microorganisms. 2026 Mar 9;14(3):606. doi: 10.3390/microorganisms14030606 (PMC13029032; doi:10.3390/microorganisms14030606)
Supplement: Supplementary file 1 [file microorganisms-14-00606-s001.zip › microorganisms-4172974-supplementary.pdf]

<u>**Supplementary materials:**</u>

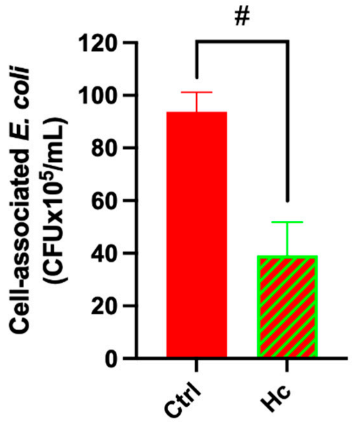

Supplementary Figure S1. Effect of pre-colonization with Hc on *E. coli* (clinical isolate) associated to T24 urothelial cells monolayer. The graph shows the mean CFUx10^5/ml ± SEM of *E. coli* associated to urothelial cells. The data reported are from n = 3 independent experiments. Statistical analysis was performed using unpaired Student's *t*-test. $^{\#}\ p < 0.05$.

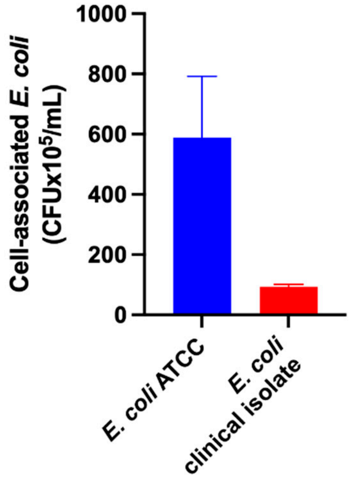

Supplementary Figure S2. *E. coli* association capacity to T24 urothelial cells monolayer. The graph shows the mean CFUx10^5/ml ± SEM of *E. coli* (ATCC strain and clinical isolate) associated to urothelial cells. The data reported are from n = 4 independent experiments (*E. coli* ATCC) and n = 3 independent experiments (*E. coli* clinical isolate). Statistical analysis was performed using unpaired Student's *t*-test.

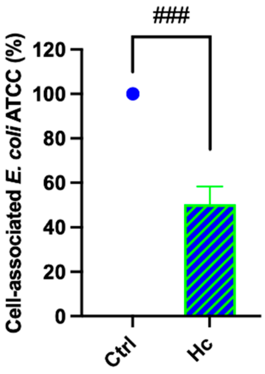

Supplementary Figure S3. Effect of pre-colonization with Hc on *E. coli* ATCC strain associated to T24 urothelial cells monolayer. The graph shows the mean % of *E. coli* associated to urothelial cells. The data reported are from n = 4 independent experiments. Statistical analysis was performed using unpaired Student's *t*-test. ### $p < 0.001$.
